# Cost-effectiveness analysis of tislelizumab plus chemotherapy as the first-line treatment for advanced or metastatic oesophageal squamous cell carcinoma in China

**DOI:** 10.1371/journal.pone.0302961

**Published:** 2024-05-15

**Authors:** Chaoneng He, Xiufang Mi, Gaoqi Xu, Xinglu Xu, Wenxiu Xin, Like Zhong, Junfeng Zhu, Qi Shu, Luo Fang, Haiying Ding

**Affiliations:** 1 School of Pharmaceutical Sciences, Zhejiang Chinese Medical University, Hangzhou, China; 2 Department of Pharmacy, Zhejiang Cancer Hospital, Hangzhou Institute of Medicine (HIM), Chinese Academy of Sciences, Hangzhou, China; 3 College of Pharmaceutical Sciences, Zhejiang University, Hangzhou, China; Affiliated Hospital of Nantong University, CHINA

## Abstract

**Objective:**

We aimed to investigate the cost-effectiveness of tislelizumab plus chemotherapy compared to chemotherapy alone as a first-line treatment for advanced or metastatic oesophageal squamous cell carcinoma (OSCC).

**Methods:**

A partitioned survival model was developed to evaluate the cost-effectiveness of tislelizumab plus chemotherapy versus chemotherapy alone in patients with advanced or metastatic OSCC over a 10-year lifetime horizon from the perspective of the Chinese healthcare system. Costs and utilities were derived from the drug procurement platform and published literature. The model outcomes comprised of costs, quality-adjusted life-years (QALYs), and incremental cost-effectiveness ratio (ICER). One-way and probabilistic sensitivity analyses were conducted to address uncertainty and ensure the robustness of the model.

**Results:**

Tislelizumab plus chemotherapy yielded an additional 0.337 QALYs and incremental costs of $7,117.007 compared with placebo plus chemotherapy, generating an ICER of $21,116.75 per QALY, which was between 1 time ($12,674.89/QALY) and 3 times GDP ($38,024.67/QALY) per capita. In one-way sensitivity analysis, the ICER is most affected by the cost of oxaliplatin, paclitaxel and tislelizumab. In the probabilistic sensitivity analysis, when the willingness-to-pay threshold was set as 1 or 3 times GDP per capita, the probability of tislelizumab plus chemotherapy being cost-effective was 1% and 100%, respectively.

**Conclusion:**

Tislelizumab plus chemotherapy was probably cost-effective compared with chemotherapy alone as the first-line treatment for advanced or metastatic OSCC in China.

## Introduction

Oesophageal cancer is a prevalent malignant tumor of the digestive system worldwide, ranking seventh in incidence (604,000 new cases) and sixth in overall mortality (544,000 deaths) [1]. Oesophageal squamous cell carcinoma (OSCC) is the most common histologic type of oesophageal cancers, accounting for 85.79% of all cases in China [2,3]. The disease burden of oesophageal cancer is particularly significant in China [1,4], the disability-adjusted life years (DALYs) caused by oesophageal cancer ranks fourth among all cancers [5]. Advanced OSCC has a poor prognosis, with a 5-year survival rate of approximately 15–25% [6]. The efficacy of conventional chemotherapy regimens for advanced OSCC is limited, with less than one year of overall survival for patients treated with first-line cisplatin in combination with paclitaxel or fluorouracil regimens [6–8].

With the recent advancements in immune checkpoint inhibitors (ICIs), there are now more treatment options available for advanced or metastatic OSCC [7,9–11]. It has been demonstrated that adding programmed death-1 (PD-1) inhibitors to standard chemotherapy can improve survival rates while maintaining manageable toxicity profiles [12–17]. According to the results of the RATIONALE-306 clinical trial, Tislelizumab, a PD-1 inhibitor, has shown superior survival benefits when used in combination with chemotherapy compared to chemotherapy alone in patients with advanced or metastatic OSCC (Median OS 17.2 vs. 10.6 months; stratified hazard ratio 0.66) [18]. And tislelizumab plus chemotherapy was recommended as the first-line treatment for advanced or metastatic OSCC by the Chinese Society of Clinical Oncology (CSCO) guideline [19].

While tislelizumab demonstrates clinical efficacy in treating advanced or metastatic OSCC, its economic burden cannot be overlooked. This study aims to compare the cost-effectiveness of tislelizumab plus chemotherapy versus chemotherapy alone as first-line treatment for advanced or metastatic OSCC from the perspective of the Chinese healthcare system.

## Materials and methods

### Population and treatments

The target population and treatment regimens in this study were consistent with those of the RATIONALE-306 trial (NCT03783442). Patients aged 18 years or older with unresectable, locally advanced, recurrent or metastatic OSCC (regardless of PD-L1 expression), Eastern Cooperative Oncology Group (ECOG) performance status of 0–1, and measurable or evaluable disease according to Response Evaluation Criteria in Solid Tumors (version 1.1) were enrolled. Included patients were randomly assigned in a 1:1 ratio to receive tislelizumab 200 mg or placebo administered intravenously every three weeks on day one, in combination with a chemotherapy doublet selected by the investigator. The doublet regimen included a platinum agent (cisplatin 60–80 mg/m² intravenously on day one or oxaliplatin 130 mg/m² intravenously on day one) combine with fluorouracil (750–800 mg/m² intravenously on days one to five) or capecitabine (1000 mg/m² orally twice daily on days one to fourteen), or paclitaxel (175mg/m² intravenously on day one).

### Model structure

**Fig 1:**
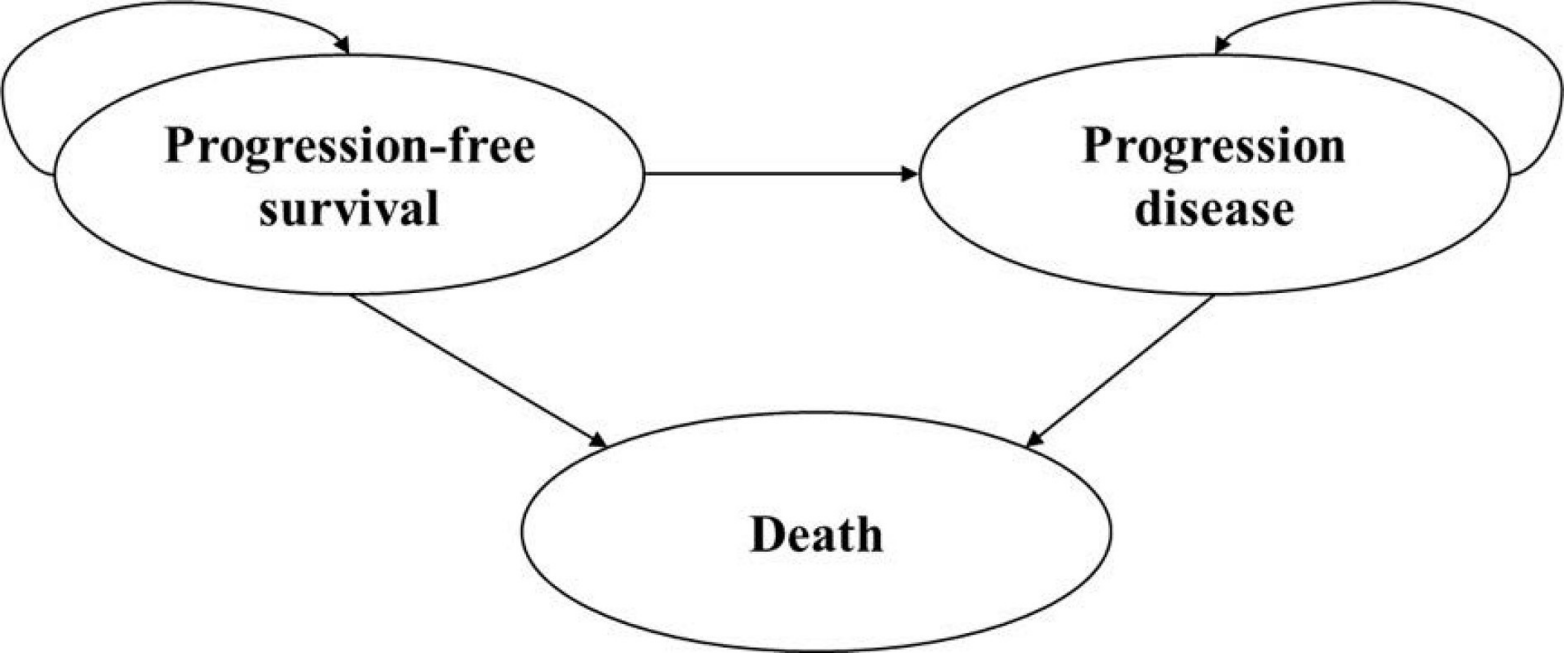
Three main health states of partitioned survival model.

A partitioned survival model was developed in Microsoft Excel to assess the cost-effectiveness of tislelizumab plus chemotherapy compared to chemotherapy alone in patients with advanced or metastatic OSCC, from the perspective of the Chinese healthcare system. The model simulated three health states: progression-free survival (PFS), disease progression (PD), and death; it is assumed that all patients enter the model in PFS state, each patient can transition from one state to another or remain unchanged within each cycle, the PD state cannot revert to the PFS state, and both the PFS state and PD state may transition to the death (**Fig 1**). The proportion of survival patients in each cycle (3-week cycle) was estimated by the area under the OS curve, and the proportion survived in PFS state was estimated by the area under the PFS curve. The proportion survived in PD state was estimated by the difference between the OS and PFS curves [20]. The proportion of patients in each health state can be directly derived from the Kaplan-Meier (K-M) curves in RATIONALE-306 trial. The cycle length was set to 3 weeks, while the time horizon was extrapolated to 10 years. The model outcomes included cost, quality-adjusted life-years (QALYs), and incremental cost-effectiveness ratio (ICER). Both costs and effectiveness are discounted at 5% annually as recommended by China guidelines for pharmacoeconomic evaluations [21]. The willingness-to-pay (WTP) threshold was set at 1or 3 times Chinese GDP per capita in 2022 as recommended by the World Health Organization (WHO) [22]. If ICER<1 time GDP per capita, the intervention strategy is totally cost-effective; if ICER is between 1 time and 3 times GDP per capita, the intervention strategy is probably cost-effective; if ICER > 3 times GDP per capita, the intervention strategy is considered not cost-effective.

### Clinical data and survival estimates

Safety and efficacy data were obtained from the results of the RATIONALE-306 trial. Firstly, K-M curves reported in the RATIONALE-306 trial were digitized using WebPlotDigitizer (https://apps.automeris.io/wpd/index.zh_CN.html). Subsequently, parametric distributions including exponential, gompertz, weibull, log-logistic and log-normal were fitted to the extracted data through the “gee” package in R software. The optimal distribution was determined by evaluating Akaike’s information criterion (AIC) and Bayesian information criterion (BIC). Both OS and PFS data were best fit with the log-logistic distribution, as evidenced by AIC, BIC.

The original and reconstructed K-M curves are presented in **S1 Fig**. Validation plots and survival distributions can be found in **S2 and S3 Figs**, respectively. Estimated parameters and goodness of fit for each survival model are reported in **S1 Table**.

### Cost and utility estimates

Only direct medical costs were considered in the model, including costs of drug acquisition, laboratory tests and radiological examinations, routine follow-up, the management of adverse events (AEs) and terminal care. All costs were converted into United States dollars with an exchange rate of 1 US dollar = 7.05 Chinese yuan (average exchange rate in 2023). The drug prices were derived from drug procurement platform (med.ybj.zj.gov.cn). To determine chemotherapy dosages and expenses, we assumed an average height of 165cm, weight of 65kg, and body surface area (BSA) of 1.72m^2^ [8]. Our model only involved severe adverse events rated as grade≥3 that were reported in at least 5% of patients in the RATIONALE-306 trial. The subsequent treatment after tislelizumab plus chemotherapy or chemotherapy alone assumes single-agent chemotherapy (e.g. docetaxel, paclitaxel or irinotecan) as recommended in the CSCO and NCCN guidelines [10,19]. Other costs were derived from previously published literature and have been recalculated using the average exchange rates for 2023 [8,23–25].

The health state utility values utilized in our model were obtained from previously published literature since quality of life for patients in the RATIONALE-306 trial was unavailable. Specifically, the utility value for progression-free survival (PFS) and disease progression (PD) in patients with advanced or metastatic OSCC were determined to be 0.75 and 0.60, respectively [23].

All the cost-related parameters and utility-related parameters were shown in **Table 1**.

**Table 1:** Model input parameters.

Parameter	Base case	Range		Distribution	Source
		Low	High		
**Costs input ($)**					
Tislelizumab (100mg)	195.39	156.31	234.47	Gamma	drug procurement platform
Cisplatin (100mg)	8.61	3.75	22.41	Gamma	
Oxaliplatin (50mg)	23.99	6.64	297.87	Gamma	
5-fluorouracil (250mg)	20.71	12.38	21.13	Gamma	
Capecitabine (500mg)	0.76	0.27	3.90	Gamma	
Paclitaxel (30mg)	15.34	7.89	99.26	Gamma	
Docetaxel(20mg)	7.52	4.26	129.08	Gamma	
Irinotecan(40mg)	69.24	7.94	87.30	Gamma	
Laboratory tests and radiological examinations per cycle	284.69	227.75	341.63	Gamma	[26]
Routine follow-up cost per cycle	29.51	23.61	35.41	Gamma	[27]
Terminal care in end-of-life	762.72	610.18	915.26	Gamma	[27]
Management cost of Anaemia	263.27	210.62	315.92	Gamma	[25]
Management cost of decreased white blood cell count	243.39	194.71	292.07	Gamma	[24]
Management cost of decreased neutrophil count	228.51	182.81	274.21	Gamma	[25]
Management cost of neutropaenia	228.51	182.81	274.21	Gamma	[25]
Management cost of hypokalaemia	7.82	6.26	9.38	Gamma	[23]
Management cost of hyponatraemia	11.54	9.23	13.85	Gamma	[23]
**Risk of adverse events in tislelizumab plus chemotherapy group (grade ≥3)**					
Anaemia	0.15	0.12	0.18	Beta	[18]
Decreased white blood cell count	0.11	0.09	0.13	Beta	[18]
Decreased neutrophil count	0.31	0.25	0.37	Beta	[18]
Neutropaenia	0.07	0.06	0.08	Beta	[18]
Hypokalaemia	0.06	0.05	0.07	Beta	[18]
Hyponatraemia	0.07	0.06	0.08	Beta	[18]
**Risk of adverse events in placebo plus chemotherapy group (grade ≥3)**					
Anaemia	0.13	0.10	0.16	Beta	[18]
Decreased white blood cell count	0.16	0.13	0.19	Beta	[18]
Decreased neutrophil count	0.33	0.26	0.40	Beta	[18]
Neutropaenia	0.10	0.08	0.12	Beta	[18]
Hypokalaemia	0.03	0.02	0.04	Beta	[18]
Hyponatraemia	0.03	0.02	0.04	Beta	[18]
**Health utility**					
Utility of PFS	0.75	0.60	0.90	Beta	[8]
Utility of PD	0.60	0.48	0.72	Beta	[8]
**Disutility due to AEs**					
Anemia	0.07	0.06	0.08	Beta	[28]
Decreased white blood cell count	0.20	0.16	0.24	Beta	[28]
Decreased neutrophil count	0.09	0.06	0.11	Beta	[29]
Neutropenia	0.20	0.16	0.24	Beta	[8]
Hypokalaemia	0.03	0.02	0.04	Beta	[8]
Hyponatraemia	0.03	0.02	0.04	Beta	[8]
**Discount rate**	0.05	0.00	0.08	Beta	[30]
**Body surface area (m** ^**2**^ **)**	1.72	1.38	2.06	Gamma	[8]

PFS, progression-free survival; PD, progressed disease.

### Sensitivity analysis

One-way deterministic sensitivity analysis (DSA) and probabilistic sensitivity analysis (PSA) were performed to assess the robustness of the model. In DSA, drug prices were adjusted within the range of the purchase price, the cost of tislelizumab was subject to a ± 20% adjustment range because there was only one purchase price, the discount rate varied from 0 to 8% [30], and ranges for other parameters were assumed to be reasonable ranges (±20%) referring to previous publications since 95% confidence intervals (CI) was not available [8,23]. The impact of each input parameter on the model results was shown in the tornado diagram. We performed 1,000 Monte Carlo simulations to assess the stability in model results when all parameters randomly varied simultaneously within the preset distribution [31]. The results of PSA were presented in the form of scatter plots and cost-effectiveness acceptability (CEAC) curves. The ranges and distributions of input parameters were shown in **Table 1.**

## Results

### Base-case analysis

The results of the base-case analysis were shown in **Table 2**. Over a 10-year horizon, tislelizumab plus chemotherapy yielded an additional 0.337 QALYs and incremental costs of $7,117.007 compared with placebo plus chemotherapy, generating an ICER of $21,116.75 per QALY, which was between 1 time ($12,674.89/QALY) and 3 times GDP ($38,024.67/QALY) per capita. The base-case result indicated that tislelizumab plus chemotherapy treatment was probably cost-effective as the first-line treatment for advanced or metastatic OSCC in China.

**Table 2:** Base-case results.

	Cost ($)	QALYs	Incremental cost	Incremental QALYs	ICER
Tislelizumab plus chemotherapy	106,466.407	1.334	7,117.007	0.337	21,116.75
Placebo plus chemotherapy	99,349.400	0.997	-	-	-

QALYs, quality-adjusted life years; ICER, incremental cost-effectiveness ratio.

### Sensitivity analysis

**Fig 2:**
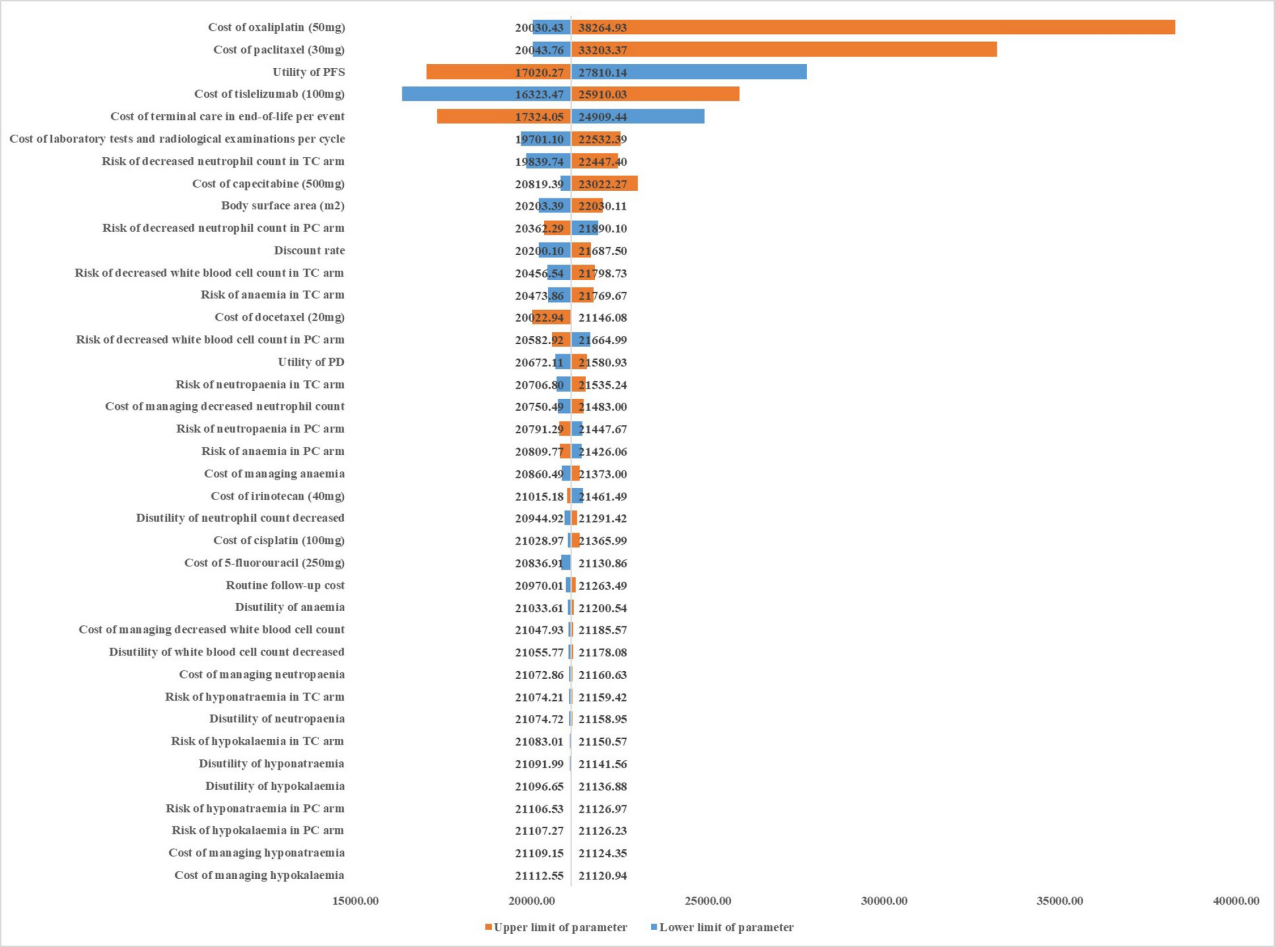
Tornado diagram of one-way sensitivity analyses of tislelizumab plus chemotherapy vs chemotherapy alone. PFS, progression-free survival; PD, progression disease.

The impact of variations in each of parameters on the ICER has been presented in tornado diagrams (**Fig 2**). The cost of oxaliplatin has the greatest impact on the ICER followed by costs of paclitaxel and tislelizumab. Among all the parameters, the price variation of oxaliplatin was the only factor that could be the reason why tislelizumab combination chemotherapy was not cost-effective, and when the price of oxaliplatin was at the upper limit ($297.87/50mg), the ICER of tislelizumab combination chemotherapy was $38,264.93/QALY, which was higher than 3 times the GDP per capita ($38,024.67/QALY).

**Fig 3:**
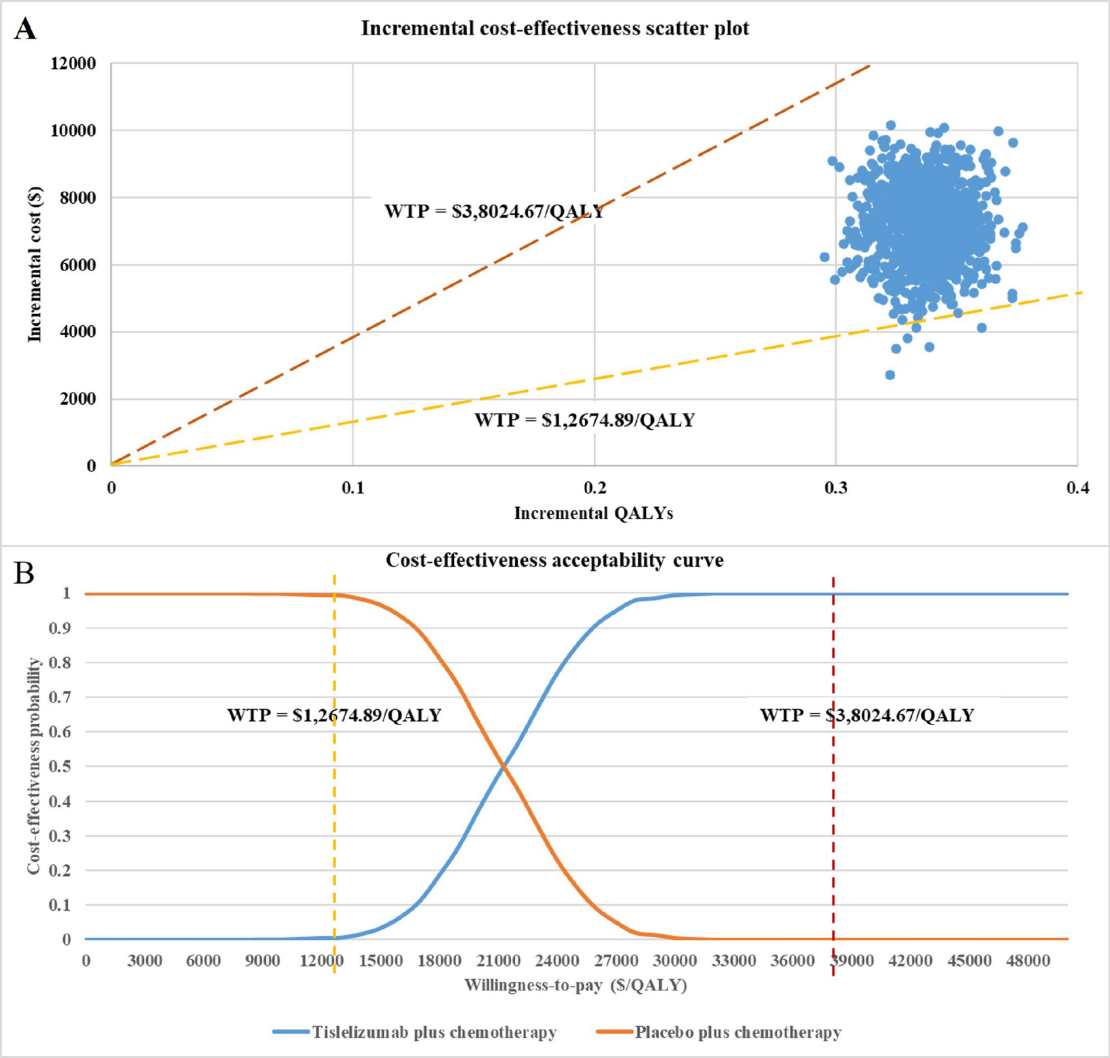
Probabilistic sensitivity analysis. (A) Incremental cost-effectiveness scatter plot; (B) Cost-effectiveness acceptability curve.

As the results of 1,000 Monte Carlo simulations shown in **Fig 3A**, a small fraction of the scatter points falls in the fourth quadrant, implying that compared to chemotherapy alone, tislelizumab in combination with chemotherapy is a dominant option. The remaining scatter points of ICER which fall in the first quadrant were all distributed below the line of the WTP threshold which was set as 3 times Chinese GDP per capita ($38,024.67/QALY), but only a tiny fraction of them distributed below the willing-to-pay threshold line of 1 time Chinese GDP per capita ($12,674.89 per QALY). According to the results of the CEAC shown in **Fig 3B**, the probability of tislelizumab plus chemotherapy treatment being cost-effective as the first-line treatment for advanced or metastatic OSCC in China was 1% and 100%, when the WTP threshold was set as 1 or 3 times GDP per capita, respectively.

## Discussion

The cost-effectiveness of tislelizumab as a first-line treatment for patients with advanced or metastatic OSCC was assessed from the perspective of the Chinese healthcare system in this study. The results showed that the ICER of tislelizumab chemotherapy plus chemotherapy compared with placebo plus chemotherapy was $21,116.75/QALY, which was between 1 time ($12,510.66/QALY) and 3 times GDP ($38,024.67/QALY) per capita. Our study demonstrated that tislelizumab and chemotherapy combination may be a cost-effective treatment strategy considering the current state of Chinese economy. The results provide data for decision-making in treatment of patients with OSCC, and serve as a reference for medical insurance access.

The CSCO guideline recommend seven PD-1 inhibitors for the first-line treatment of OSCC. The economics of PD-1 inhibitors for OSCC treatment are also receiving increasing attention. Several cost-effective results of PD-1 inhibitors combined with chemotherapy in OSCC treatment have been published [8,23,31–40]. A cost-effectiveness analysis was conducted to compare different PD-1 inhibitors in combination with chemotherapy for the first-line treatment of advanced OSCC in China [8]. Moreover, two studies investigated the cost-effectiveness of tislelizumab in the first-line treatment of oesophageal squamous carcinoma, which were similar to the design of our study [41,42]. Lu S. et al.’ study [42] used the Markov model, while our study used the partitioned survival model. The study of Zhou C. et al. [41] used a partitioned survival model, but there were some limitations in the methodology. Firstly, the chemotherapy regimen of the first-line treatment combined with tislelizumab only included cisplatin plus paclitaxel, which was inconsistent with the design of the RATIONALE-306 clinical trial. In the clinical trial, the chemotherapy regimen for the first-line treatment was platinum (cisplatin/oxaliplatin) plus fluoropyrimidine (5-fluorouracil/capecitabine) or paclitaxel [18]. It is necessary to include oxaliplatin in model simulation since oxaliplatin is preferred over cisplatin due to its lower toxicity according to the NCCN Guideline [10]. Furthermore, fluoropyrimidine also occupied a crucial position in the clinical practice in treating oesophageal cancer patients in China. Our study addressed this issue by selecting the initial treatment regimen based on the clinical trial by including oxaliplatin and fluorouracil. Secondly, in Zhou C. et al’ study, the subsequent treatment regimen assumed cisplatin plus paclitaxel, which is not recommended as a second-line treatment in either the CSCO or the NCCN guidelines [10,19]. Our study optimised the assumption by selecting a second-line regimen based on the recommendations of both guidelines, ultimately choosing a single-agent chemotherapy (e.g. docetaxel, paclitaxel or irinotecan).

In the one-way sensitivity analysis, the prices of oxaliplatin, paclitaxel, and tislelizumab were identified as the top three parameters that affect the results of the partitioned survival model. The great impact of the prices of oxaliplatin and paclitaxel on the results might due to the wide range of prices between generic and original drugs. The low price for tislelizumab resulted in the tislelizumab combination strategy being cost-effective and advantageous in treatment of patients with OSCC. During the Chinese national drug pricing negotiations in 2021 and 2022, the price of tislelizumab was reduced by 80% and 33%, respectively. Chinese national drug pricing negotiations is an effective way to lower the drug prices and make the innovative drug more cost-effective. The drugs negotiated during the 2023 agreement period benefited over 210 million patients and result in a reduction of over 200 billion yuan in financial burden for patients. This study also confirms the importance of China’s national drug pricing negotiation policy, which is significant in enhancing drug supply and reducing the medical burden, the further reduction of the price of tislelizumab is a major measure to improve its economy.

There are several limitations in the study. First, our cost-effectiveness analysis was based on the RATIONALE-306 clinical trial, where treatment options and costs were as close to the trial design as possible and were not as complex and variable as real-world clinical treatments. Second, since the RATIONALE-306 trial did not publish data for quality of life, the utilities in this analysis were derived from previously published literature, which may not reflect the true health status of the patients treated. Third, the fitting extrapolation method based on the parameter distribution of the survival curves used in the study tends to ignored the short observation period of clinical study and failed to identify non-disease-related deaths, which would make the extrapolated survival curve inconsistent with reality.

## Conclusions

From the perspective of the Chinese healthcare system, tislelizumab plus chemotherapy was probably cost-effective compared with chemotherapy alone as the first-line treatment for advanced or metastatic OSCC in China. Our results could provide new data for decision-making in treatment of patients with OSCC and bring more confidence to the implementation of drug price negotiation policy in China. However, real-world studies are still necessary to validate the efficacy, safety and economics of tislelizumab compared with other alternative strategies.

## Supporting information

S1 Fig Original and reconstructed Kaplan–Meier curves. (A) Original overall survival curves from the RATIONALE-306 trial. (B) Reconstructed overall survival curves. (C) Original progression free survival curves from the RATIONALE-306 trial. (D) Reconstructed progression free survival curves.
(TIF)

S2 Fig The parametric progression-free survival curves of tislelizumab plus chemotherapy vs chemotherapy in the RATIONALE-306 trial. (TIF)

S3 Fig The parametric overall survival curves of tislelizumab plus chemotherapy vs chemotherapy in the RATIONALE-306 Trial. (TIF)

S1 Table Estimated parameters and goodness of fit from each survival model. (XLSX)
