# Supplementary figures and images for: Cost-effectiveness analysis of tislelizumab plus chemotherapy as the first-line treatment for advanced or metastatic oesophageal squamous cell carcinoma in China
Source: PLoS One. 2024 May 15;19(5):e0302961. doi: 10.1371/journal.pone.0302961 (PMC11095747; doi:10.1371/journal.pone.0302961)

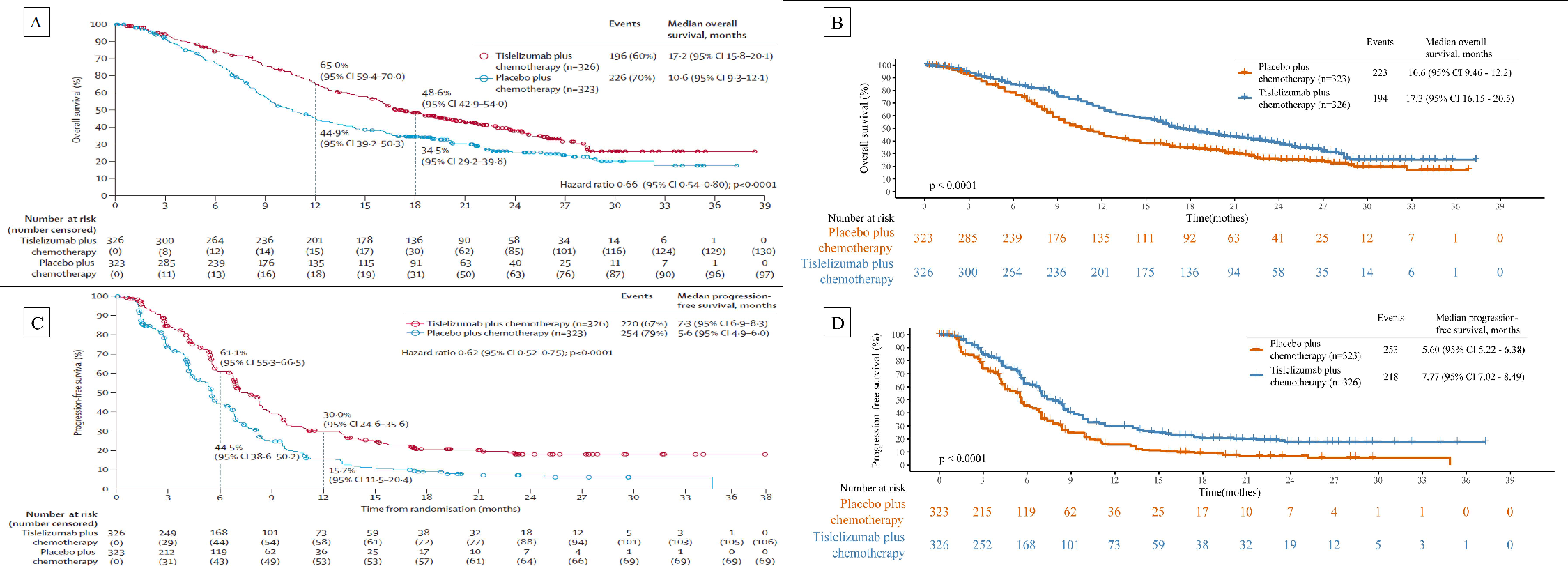

Supplement: S1 Fig — (A) Original overall survival curves from the RATIONALE-306 trial. (B) Reconstructed overall survival curves. (C) Original progression free survival curves from the RATIONALE-306 trial. (D) Reconstructed progression free survival curves. (TIF) [file pone.0302961.s001.tif]

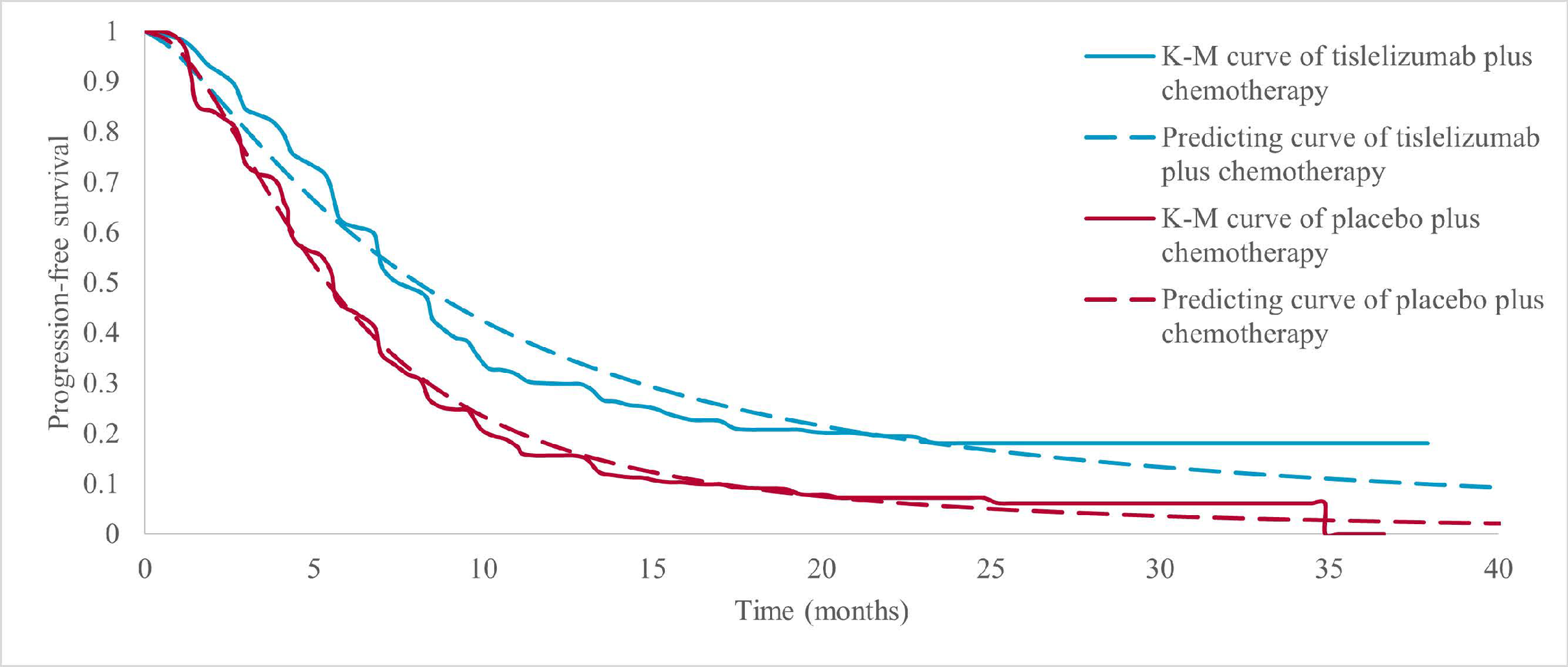

Supplement: S2 Fig — (TIF) [file pone.0302961.s002.tif]

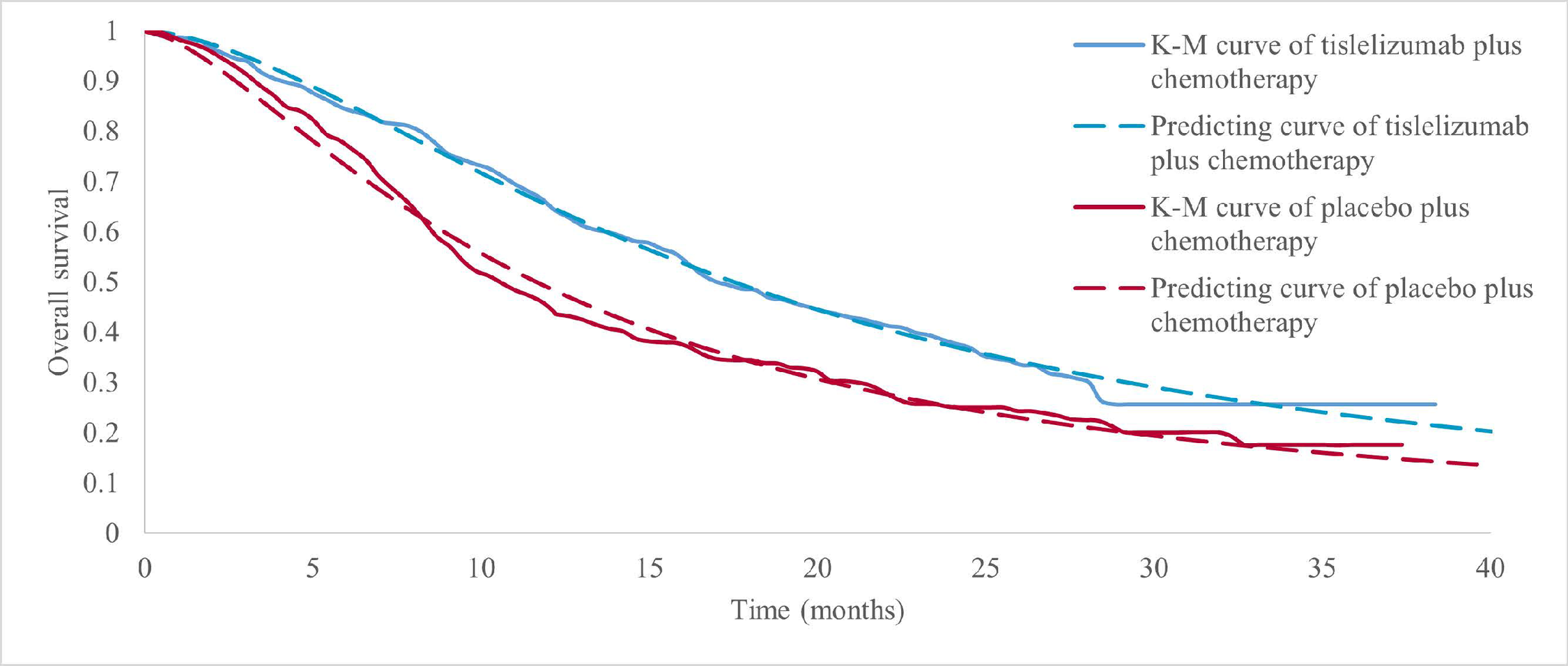

Supplement: S3 Fig — (TIF) [file pone.0302961.s003.tif]
